# The role of intimate partner violence and other health-related social factors on postpartum common mental disorders: a survey-based structural equation modeling analysis

**DOI:** 10.1186/1471-2458-14-427

**Published:** 2014-05-05

**Authors:** Michael Eduardo Reichenheim, Claudia Leite Moraes, Claudia Souza Lopes, Gustavo Lobato

**Affiliations:** 1 Department of Epidemiology, Institute of Social Medicine (IMS), Rio de Janeiro State University (UERJ), Rua São Francisco Xavier 524, 7° andar, Rio de Janeiro, RJ 20550-013, Brazil; 2 Family Health Master Program, Estácio de Sá University, Rua Riachuelo, 27, Rio de Janeiro, RJ 20230-010, Brazil; 3 Fernandes Figueira Institute, Oswaldo Cruz Foundation (FIOCRUZ), Avenida Rui Barbosa, 716 – 3° Andar, Rio de Janeiro, RJ 22250-020, Brazil

**Keywords:** Intimate partner violence, Social determinants of health, Postpartum common mental disorder, Mental health, Structural equation model

## Abstract

**Background:**

Although studies suggest the relevance of intimate partner violence (IPV) and other health-related social characteristics as risk factors for postpartum mental health, literature lacks evidence about how these are effectively connected. This study thus aims to explore how socio-economic position, maternal age, household and marital arrangements, general stressors, alcohol misuse and illicit drug abuse, and especially psychological and physical IPV relate in a framework leading to postpartum common mental disorder (CMD).

**Methods:**

The study was carried out in five primary health care units of Rio de Janeiro, Brazil, and included 810 randomly selected mothers of children up to five postpartum months waiting for pediatric visits. The postulated pathways between exposures and outcome were based on literature evidence and were further examined using structural equation models.

**Results:**

Direct pathways to postpartum CMD arose from a latent variable depicting socio-economic position, a general stressors score, and both IPV variables. Notably, the effect of psychological IPV on postpartum CMD ran partly through physical IPV. The effect of teenage pregnancy, conjugal instability and maternal burden apparently happens solely through substance use, be it alcohol misuse, illicit drug abuse or both in tandem. Moreover, the effect of the latter on CMD seems to be entirely mediated through both types of IPV.

**Conclusion:**

Although the theoretical model underlying the analysis still requires in-depth detailing, results of this study may have shed some light on the role of both psychological and physical IPV as part of an intricate network of events leading to postpartum CMD. Health initiatives may want to make use of this knowledge when designing preventive and intervention approaches.

## Background

Although largely neglected by global health policies, mental disorders are estimated to account for 14% of the global burden of disease [1]. Beyond severe cases of mental health problems, there is a whole range of mental disorders showing up in basic health services, be they in patients in search of health care for physical illnesses or complaints, or patients seeking contact with services for preventive or follow up care (e.g., pre or postnatal care). Expected as a set to tap an array of non-psychotic disorders, these recurrent complaints have been brand-named common mental disorders (CMD) or psychological distress in the late 1980s and since used in many papers [2-4]. A specific collection of symptoms most often includes depression, anxiety and somatic complaints [2,5]. CMD’s comprise 90% of all of the psychiatric morbidities and, according to WHO, need to be regarded as a major public health concern [6].

Population-based studies have shown that CMD are more prevalent among the poor, unemployed, persons with low social support or who have experienced stressful life events, alcohol and drug consumers, and those with less years of schooling [7-9]. Gender differences are also consistent in the literature [10,11], with anxiety and mood disorders being approximately twice as common in women as in men [4], regardless if occurring in higher or lower income countries [12]. However, such gender differential rates are strongly age-related; the greatest differences occur in adult life, with no reported differences in childhood and few in the elderly [12,13]. It has also been recognized that CMD have important consequences not only to women, but also to the development and wellbeing of their children [14-16].

A comprehensive approach is required for understanding the higher prevalence of CMD among women. Recently, the role of genetic, biological and psychosocial characteristics as important determinants of women’s mental health have been reinforced [6,13,14,17], and better explanations regarding these intricate relations have been pursued [18].

Consistent evidence across different cultures and countries suggests that social and economic characteristics play an important role in postpartum CMD [19,20]. Several studies also suggest that the effect of socio-economic position on women’s mental health is shaped by the ensuing reproductive circumstances [8], stressful life experiences [21], substance consumption [8], and features related to the prevailing marital relationship [21].

The relationship between life events and women’s mental health in the postpartum period is also well established [19,20,22] and research shows that stressful experiences tend to precede substance consumption and intimate partner violence (IPV) in the context of women’s mental disabilities [21,23]. Similarly, early (youth/adolescent) pregnancy, unstable relationships, and the burden resulting from several young offspring to care for seem to converge in increasing the risk of developing depressive and anxiety symptoms after birth [14,19,20,24]. There is also evidence that these events are important predictors of IPV during pregnancy and postpartum [25] and are commonly associate with misuse or abuse of psychoactive substances [21,23]. Along the pathways leading to mental disorders among women, some studies show that the effect of alcohol misuse or illicit drug abuse are at least partially mediated by IPV [21,26]. It should be noticed that the available evidence consistently portrays violence as an end-point consequence (outcome) in the process, suggesting IPV as a proximal path leading up to CMD.

There is also a substantial literature focusing chiefly on violence between couples as a risk factor to CMD among women [27-30], including mood disorders during pregnancy and the postpartum period [14,31-34]. Yet, there is not much evidence on the role played by escalating acts of psychological and physical partner abuse on CMD following childbirth. Some nuances of these intricate relationships remain unclear, as psychological and physical abuse have been generally treated separately in epidemiologic studies, therefore hindering a better understanding of how these two dimensions effectively associate amid a complex system holding other important events. This is hardly surprising since most of the studies on the subject have employed traditional analyses [22,35-42], which preclude explicitly addressing mediating processes.

Putting it all together, it is clear that research on the relationship between IPV and women’s poor mental health following childbirth still falls short on providing a comprehensive account [27]. An issue still requiring light concerns the pathways by which socio-demographic characteristics, stressful events, substance consumption and IPV relate to each other, and in tandem, to the lack of psychological well-being in the postpartum period. It is true that some evidence is available, but the focus has mostly been on specific relationships, failing therefore to offer a more inclusive and organic perspective. In an attempt to redress this gap, the present study focuses on how IPV during pregnancy relates to CMD among women in the postpartum period, amid other biopsychosocial and health-related social covariates forming a complex interconnected framework. The analysis uses structural equation models, thus allowing the simultaneous evaluation of direct and indirect effects of antecedent and mediating variables.

## Methods

### Participants

The sample included randomly selected mothers of children under 5 months of age who were waiting to be consulted in five large public primary health care (PHC) facilities of Rio de Janeiro, Brazil. These PHC units comprised a convenience sample of health centers located throughout the city and serving mostly health care users of low to moderate income and education living in adjacent areas. This broad geographical distribution sought a representation of different sociocultural contexts represented in the study population. In addition, these health facilities provided assistance through spontaneous demand or scheduled consultations in general practice, pediatrics, gynecology and obstetrics. Minor surgical procedures, vaccinations and general actions for health promotion were also offered. Each unit performed, on average, 1,300 pediatric consultations per month at the time of data collection.

Data collection occurred from January to July 2007. On every day shift, a list of children was prepared prior to the consultations. Next, a draw was carried out in order to determine which mother would be interviewed first. Following each interview, the list was rerun, and another draw was taken. Face-to-face interviews of c. 45 minutes involving a multi-dimensional questionnaire of 368 questions were conducted by previously trained female health professionals under the supervision of two field coordinators. Given some shared research purposes [25,43-51], women were considered ineligible when experiencing less than one month of intimate relationship with a partner during pregnancy or the postpartum period, if there was an absolute contraindication for breastfeeding, and if they had given birth to twins. Out of 853 women invited to participate in the study, 18 (2.1%) were ineligible and, from the remaining 835, 24 (2.9%) refused to participate. Only one woman had missing data in one of the variables to be modeled. The effective study sample thus included 810 women who were interviewed in a reserved area without the presence of anyone but the interviewer, after signing an informed consent. Anonymity and confidentiality were completely assured. Women were informed about available health care units providing special services and hostels for cases of domestic violence in various parts of the city of Rio de Janeiro, regardless of whether they were identified as victims or not. The study was approved by the Research Ethics Committee of Rio de Janeiro Municipal Health Department in conformity with the principles embodied in the declaration of Helsinki.

### Variables and measurements

**Figure 1:**
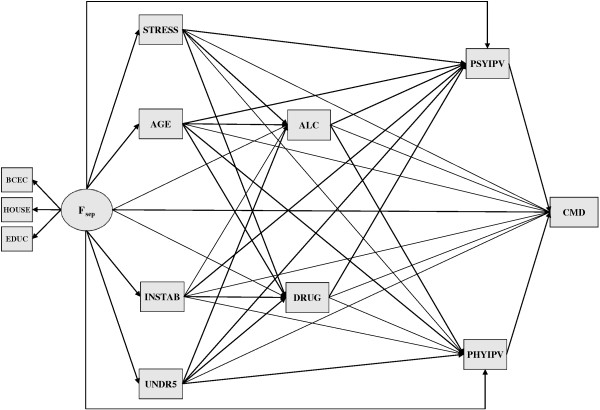
**Direct acyclic graph depicting the propositional model.** Variable definitions and mnemonics used in the DAG are provided in Table 1.

Figure 1 presents the direct acyclic graph (DAG) portraying the initial ‘propositional’ model put to testing. Derived from the literature reviewed in the Introduction, this model specifies five domains comprising socio-economic position, stressors, substance intake, intimate partner violence, and common mental disorders. Structural paths move from left to right respecting the conjectured hierarchical sequence of events. Variable details are provided next, including definitions and respective measurement tools (where applicable). Table 1 provides a summary of the coding according to how the variables were used in the main analysis using SEM.

**Table 1:** Variables, instruments/composition and scaling/scoring (as used in the main structural equation models)

**Variable**	**(mnemonic)**	**Instrument/composition**	**Scaling**	**Scoring (categorization)**
Brazilian Criterion of Economic Classification	(BCEC)	• Household assets: TV set, DVD set, radio, vacuum cleaner, washing machine, refrigerator, freezer, bathroom, car	Sum of item scores (0–34)	• Stratum A (highest) → score ≥ 25
				• Stratum B → score ≥ 17-24
				• Stratum C → score ≥ 11-16
		• Presence of maid in the household		• Stratum D → score ≥ 6-10
		• Educational status of the main breadwinner		• Stratum E (lowest) → score ≤ 5
Household conditions	(HOUSE)	• Household density: people per room	Sum of item scores (0–7)	• Maximum (best) → 7 points
		• Type of house floor		• Minimum (worst) → 0
		• Type of garbage/waste disposal system		
		• Type of sewage		
Women’s schooling achievements	(EDUC)	• Number of school years concluded	One year intervals	• Maximum → 12 years +
				• Minimum → 0
Stressful live events taking place during the latest pregnancy	(STRESS)	• Death of the partner or a close relative	Sum of item scores (0–5)	• Maximum → 5 points (events)
		• Job loss by the women		• Minimum → 0 points (events)
		• Job loss by her partner		
		• Forced eviction from home		
		• Perceived unusual financial problems		
Women’s age at the beginning of the latest pregnancy	(AGE)	• Number of years	One year intervals	• Maximum → 44 years
				• Minimum → 13 years
Marital status (proxy to the degree of conjugal instability)	(INSTAB)	• Weather woman/mother currently had a partner or not during pregnancy	Composition	• Stable → woman reporting the same spouse throughout the latest pregnancy and thereafter, conditional on the partner being the father of the current child
		• Weather woman/mother currently had a partner or not during the post natal period		
		• Weather the referred to partner was the same in both periods		• Unstable → otherwise
		• Weather partner was the father of the child		
Children under five at home (proxy to the level of women’s work load and burden)	(UNDR5)	• Simple count	Sum of item scores (0–3)	• Maximum (worst) → 3 children
				• Minimum → 1 child
Alcohol misuse during pregnancy (couple)	(ALC)	• TWEAK (*Tolerance; Worried; Eyeopener; Amnesia; K/Cut-down*) [54,55] → women	Sum of item scores (0–7) [TWEAK] (0–4) [CAGE]	Joint score (women and partner)
		• CAGE (*Cut-down; Annoyed; Guilty; Eye-opener*) [56-58] → partner		• Maximum (worst) → 10 points
				• Minimum (best) → 0 points
Illicit drugs abuse during pregnancy (couple)	(DRUGS)	• NSDUQ (the Non-Student Drugs Use Questionnaire (NSDUQ) [59] (used on women and partners)	Sum of item scores (0–4)	Joint score (women and partner)
				• Maximum (worst) → 5 points
				• Minimum (best) → 0 points
Psychological intimate partner violence occurring during pregnancy (couple)	(PSYIPV)	• CTS2 (Revised Conflict Tactics Scale) [60-62]	Sum of item scores (0–8)	Joint score (women and partner)
				• Maximum (worst) → 16 points (+ve)
				• Minimum (best) → 0 points
Physical intimate partner violence occurring during pregnancy (couple)	(PHYIPV)	• CTS2 (Revised Conflict Tactics Scale) [60-62]	Sum of item scores (0–12)	Joint score (women and partner)
				• Maximum (worst) → 24 points (+ve)
				• Minimum (best) → 0 points
Common Mental Disorders (women)	(CMD)	• SRQ-20 (Self-Reporting Questionnaire) [3,63,65,66,69]	Sum of item scores (0–20)	• Maximum (worst) → 20 points (+ve)
				• Minimum (best) → 0 points

### Socio-economic position

The family’s socio-economic position was represented through a ‘latent’ variable (factor) ‘manifested’ by three empirical variables: (1) the Brazilian Criterion of Economic Classification (BCEC); (2) an omnibus score based on key household conditions (HOUSE); and (3) women’s schooling achievements (EDUC). The BCEC is a composite index comprised of a selected basket of available household assets and the educational status of the main breadwinner [52]. As recommended, the index was categorized into five strata, ranging from the richest (stratus A) to the most disadvantaged economic group (stratus E). HOUSE is formed by four characteristics, *viz.*, availability and quality of water supply services, type of sewerage and sanitation, type of domestic refuse and garbage disposal system, and a variable seizing household density (persons/rooms). The total score ranges from 0 to 7 [53]. For descriptive purposes, the index was further discretized into three levels (7, 6 and ≤ 5). Modeled as an ordinal score (0 to 12), women’s schooling was similarly dichotomized for descriptive purposes at the cut-off point of 9 years of completed schooling, which stands for the Brazilian basic education level (equivalent to middle school in the US and O-level in the UK).

### Stressors

Four variables were selected to represent major stressor situations: stressful live events taking place during the latest pregnancy (STRESS); women’s age at the beginning of the latest pregnancy (AGE); marital status as a proxy to the degree of conjugal instability (INSTAB); and the number of children under five at home as a proxy to the level of women’s work load and burden (UNDR5).

Five closed questions/indicators were used to tap distressing life events during pregnancy, *viz.*, death of the partner or a close relative, job loss by the women, job loss by her partner, forced eviction from home, and perceived unusual financial problems. The cumulative total score (0 to 5) was used in the main analysis (modeling), but a three-level variable is presented for the purpose of descriptively highlighting the percentage of women with and without any stressor experienced during pregnancy. Age and number of children under five were used in the main analyses as continuous and ordinal variables, respectively. For descriptive purposes, the former was categorized with the aim of underlining the frequency of pregnancy among teenagers and in more mature women. Conjugal instability was defined as any situation except when the woman reported the same spouse throughout the latest pregnancy and thereafter, conditional on the partner being the father of the current child.

### Substance use

Women’s alcohol misuse (ALC) during pregnancy was measured through the TWEAK (*Tolerance; Worried; Eyeopener; Amnesia; K/Cut-down*) [54,55], while the CAGE (*Cut-down; Annoyed; Guilty; Eye-opener*), based on women’s report (by proxy), was used to measure their partners’ alcohol intake [56-58]. The Non-Student Drugs Use Questionnaire (NSDUQ) was used to identify illicit drugs abuse (DRUGS) [59], and women’s report (by proxy) employed to characterize partner’s intake of illegal substances.

Seeking the highest possible sensitivity, and in an attempt to deal with possible misreporting due to the proxy approach, both variables ALC and DRUGS were respectively modeled as ordinal scores comprised by the sum of the scores from both members of the couple. Thus, given that the total TWEAK and CAGE scores respectively varied from 0 to 7 and 0 to 4, ALC could take values from 0 to 11. Similarly, DRUGS would take values from 0 to 8 since the NSDUQ score ranges from 0 and 4. In presenting the profile of alcohol misuse, both TWEAK and CAGE scores were first dichotomized (yes/no) at the recommended cut-off points of 2 [54-58] and joined thereafter to form a four-level variable depicting whether alcohol misuse was either positive in one or both members of the couple. The same was done with NSDUQ, based on the recommended cut-off point of 1 [59].

### Intimate partner violence

A Brazilian version of the Revised Conflict Tactics Scale (CTS2) was used to gauge IPV during pregnancy [60-62]. The CTS2 comprises 78 indicators describing acts perpetrated by the respondent and reciprocally by the partner. Although these items form five sub-scales, only two were of interest in this study: psychological aggression and physical violence during pregnancy. The sub-scale on psychological IPV (PSYIPV) comprises eight dichotomous items (whether the event ever happened or not and if so, if it occurred at least once during pregnancy) relating to women as perpetrators and/or victims. Raw scores could thus range from 0 to16. Likewise, physical IPV (PHYIPV) involves 12 items, with respective score thus varying from 0 to 24. For descriptive purposes, both variables were classified in three levels (0, 1 or ≥ 2 events).

### Common mental disorders

The Self-Reporting Questionnaire (SRQ-20) was used to assess CMD [63]. The SRQ-20 was developed by the World Health Organization (WHO) for a first approach to be used in primary health care services [64]. The scale consists of twenty dichotomous items covering depression (e.g., feel unhappy, find it difficult to enjoy daily activities, feel worthless, thought of ending your life been on your mind), anxiety (e.g., sleep badly, easily frightened, hands shake, feel nervous, tense or worried) and somatization symptoms (e.g., feel tired all the time, uncomfortable feelings in your stomach). Scores thus range from 0 and 20, which implicitly increase with the degree of psychological distress. This raw score was used in the modeling process, whereas a dichotomous variable applying the recommended cut-off point of 7/8 for adult women was employed for descriptive purposes [63,65,66].

Evidence of the SRQ-20 validity accumulate since its introduction in 1980 [3]. In Brazil, it was initially studied by Mari & Williams [65,66] who compared the instrument against the criterion of the Clinical Interview Schedule (CIS) [67], showing a sensitivity of 83%, a specificity of 80%, and an area under the ROC curve of 0.90. More recently, the Brazilian version of the SRQ-20 was assessed in the elderly by Scazufca et al. [68], who showed a sensitivity of 76.1%, a specificity of 74.6%, and an area under ROC curve of 0.82. The instrument’s factor structure has been studied by Iacoponi and Mari [69] and Santos et al. [70].

### Statistical analysis

The structural equation modeling process employed Mplus’ robust weighted least squares mean and variance adjusted estimator (WLSMV) [71]. Except for variables INSTAB (2 levels) and UNDR5 (3 levels), all others were assumed as normally distributed and thus modeled accordingly. Goodness of fit was evaluated using three indices [72,73]. The Root Mean Square Error of Approximation (RMSEA) incorporates a penalty function for poor model parsimony [72,74,75]. Values under 0.06 suggest close approximate (adequate) fit, whereas values above 0.10 indicate poor fit and that the model should be rejected [71,76]. The Comparative Fit Index (CFI) and the Tucker-Lewis index (TLI) represent incremental fit indices contrasting the hypothesized model to a more restricted nested baseline model, the “null model” [72,73]. Both range from zero to one and values > 0.9 are indicative of adequate fit [77].

The model re-specification process took two sequential stages. Starting with the ‘propositional’ model shown in Figure 1, we first looked at indications of model misspecification observing suggested left out paths. To this end Modification Indices (MI) were used. A MI reflects how much the overall model chi-square decreases (improves) if a constrained parameter is freely estimated. Candidate paths involving conspicuous values (MI > 10) were then examined for the actual amount of model fit improvement and for the magnitude of the ensuing freely estimated coefficients. The decision to explore and keep new paths also followed their theoretical meaningfulness [73].

The second stage involved systematically trimming out non-significant paths, i.e., coefficient estimates with p-value > 0.05. This process adhered to the hierarchical principal advocated in the model. The trimming process thus moved from left to right, starting with all paths stemming from < *F*_*ses*_>, followed by < STRESS/AGE/INSTAB/UNDR5>; then < ALC/DRUGS>; and finally < PSYIPV/PHYIPV>. At each step, interim evaluations of MIs were carried out in search of any relevant path arising once the model had been simplified. The overall process stopped when none additional path was suggested by the MIs, while all remaining paths retained statistical significance given acceptable levels of model fit.

## Results

### Profile of the study population

The mean SRQ-20 score was 5.4 (s.d. 3.9) and 25.4% (95% CI: 22.4; 28.4) women scored positive according to the cut-off point of ≥7. Table 2 shows that according to the BCEC, almost nine in ten women belonged to the lower-middle (C) or lower socio-economic strata (D and E). This is in agreement with only one-quarter of household conditions attaining the highest score (7) that summarizes all components —water, sanitation and refuse disposal, and household density— at their best, as well as with the overall level of achieved schooling. The population comprised 27.5% teenage pregnancies, 13.5% women reporting unstable conjugal relationships, and 25.8% involved in caring for two or more children. About half referred to at least one stressful life event occurring during pregnancy (52%).

**Table 2:** Profile of the study population: univariate analysis and frequency of CMD (positive) according to variable status

**Variables**	**Univariate**		**CMD (+ve)**		
	**n**	**%**	**n**	**%**	**p-value**
Socio-economic strata (BCEC)					
*A*	15	1.9 (0.9 - 2.8)	2	13.3 (3.1 - 41.8)	0.011
*B*	81	10.0 (7.9 - 12.1)	12	14.8 (8.5 - 24.3)	
*C*	369	45.5 (42.1 - 49.0)	86	23.3 (19.2 - 27.9)	
*D*	330	40.7 (37.3 - 44.1)	100	30.3 (25.5 - 35.4)	
*E*	15	1.9 (0.9 - 2.8)	6	40.0 (18.5 - 66.0)	
Household condition (score)					
*7* (best)	199	24.6 (21.6 - 27.5)	33	16.5 (12.0 - 22.4)	< 0.001
*6*	235	29.0 (25.9 - 32.1)	49	20.8 (16.1 - 26.5)	
*≤ 5* (worse)	376	46.4 (43.0 - 49.9)	124	33.0 (28.4 – 37.9)	
Schooling (women)					
*> 9 years*	295	36.4 (33.1 - 39.7)	49	16.6 (12.7 - 21.3)	< 0.001
*≤ 9 years*	515	63.5 (60.2 - 66.9)	245	83.3 (78.6 - 87.2)	
Women’s age at the beginning of latest pregnancy					
*> 35 years*	70	8.6 (6.7 - 10.6)	9	12.3 (06.5 - 22.1)	0.003
*20 - 35 years*	517	63.8 (60.5 - 67.1)	138	24.9 (21.4 - 28.6)	
*< 20 years*	223	27.5 (24.4 - 30.6)	59	32.2 (25.8 - 39.3)	
Conjugal instability					
*No*	701	86.5 (84.2 - 88.9)	142	21.8 (18.8 - 25.1)	< 0.001
*Yes*	109	13.5 (11.1 - 15.8)	64	40.5 (33.1 - 48.4)	
Number of children under 5 years					
*One*	601	74.2 (71.2 - 77.2)	135	22.5 (19.3 - 26.0)	0.002
*Two*	173	21.4 (18.5 - 24.2)	55	31.8 (25.3 - 39.1)	
*Three*	36	4.4 (3.0 - 5.9)	16	44.4 (29.1 - 60.9)	
Stressful life events during pregnancy					
*None*	389	48.0 (44.6 - 51.4)	45	11.6 (8.7 - 15.2)	< 0.001
*One event*	281	34.7 (31.4 - 38.0)	93	33.1 (27.8 - 38.8)	
*Two or more events*	140	17.3 (14.7 - 19.9)	68	48.6 (40.4 - 56.9)	
Misuse of alcohol					
*No*	421	52.0 (48.5 - 55.4)	79	18.8 (15.3 - 22.8)	< 0.001
*Woman only*	202	24.9 (21.9 - 27.9)	59	29.2 (23.3 - 35.9)	
*Partner only*	117	14.4 (12.0 - 16.9)	43	36.8 (28.5 - 45.9)	
*Both*	70	8.6 (6.7 - 10.6)	25	35.7 (25.3 - 47.6)	
Abuse of illicit drugs					
*No*	695	85.8 (83.4 - 88.2)	154	22.2 (19.2 - 25.4)	< 0.001
*Woman only*	11	1.4 (0.6 - 2.1)	6	54.5 (25.6 - 80.7)	
*Partner only*	82	10.1 (8.0 - 12.2)	33	40.2 (30.2 - 51.2)	
*Both*	22	2.7 (1.6 - 3.8)	13	59.1 (37.7 - 77.5)	
Psychological IPV during pregnancy					
*None*	146	18.0 (15.4 - 20.7)	19	13.0 (8.4 - 19.5)	< 0.001
*One event*	77	9.5 (7.5 - 11.5)	18	23.4 (15.2 - 34.2)	
*Two or more events*	587	72.5 (69.3 – 75.4)	169	28.8 (25.3 - 32.6)	
Physical IPV during pregnancy					
*None*	504	62.2 (58.9 - 65.6)	86	17.1 (14.0 - 20.6)	< 0.001
*One event*	87	10.7 (8.6 - 12.9)	27	31.0 (22.2 - 41.6)	
*Two or more events*	219	27.0 (24.0 - 30.2)	93	42.5 (36.1 - 49.1)	

Note: Variable levels used for description are based on the scorings used in the main analysis (SEM). See Table 1.

Alcohol misuse was found in almost half of couples (48%), while 14.2% reported having used an illegal drug. Combining both, 51.6% (95% CI: 48.2 - 55.1) of couples referred to misusing any substance (not shown in Table 2). The frequency of at least one episode of IPV experienced either as perpetrator or victim by any member of the couple was high, be it of a psychological (82%) or physical (37.8%) type. While only 4% of the couples reporting an absence of psychological aggression referred to an episode of physical abuse, this figure rose to 45.2% among couples concomitantly reporting a positive psychological aggression.

Table 2 also shows the population profile according to CMD positive status. All variables are statistically significant. There is an increasing gradient of positive CMD as the situation indicated in each variable worsens.

### Main findings

The first ‘propositional’ model fitted rather poorly —RMSEA: 0.084 (95% CI: 0.073 - 0.096); CFI: 0.907 and TLI: 0.763—, while the Modification Indices suggested two important paths to be included and freely estimated (AGE → INSTAB and PSYIPV → PHYIPV). With those, the second model fared better, but beyond mere adjustment issues, several paths lacked statistical significance at the 0.05 level and were candidates for further trimming. Following the analytical procedure outlined in the Methods section, it took another seven sequential steps to settle on a ‘final’ model.

**Figure 2:**
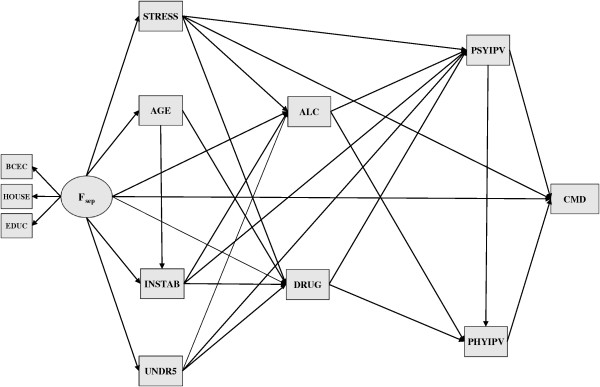
**Direct Acyclic Graph depicting the final model.** Variable definitions and mnemonics used in the DAG are provided in Table 1.

Figure 2 shows the Direct Acyclic Graph of this ninth and ‘final’ model. Respective estimates and the adjustment indices are shown in Table 3. Intimate partner violence of both types is associated to CMD, conditional on other variables in the system. As mentioned before, there is now an explicit path between psychological aggression and physical abuse. Regarding PSYIPV, its direct ‘effect’ entailing other unspecified effectors/paths is only half that found for PHYIPV. However, the total effect of PSYIPV, based on the sum of the indirect effect (obtained from the products of the component coefficients *β*_{PSYIPV → PHYIPV}_ and *β*_{PHYIPV → CMD}_) and the direct effect (*β*_{PSYIPV → CMD}_) is almost the same as that found for PHYIPV on CMD. Using a contrast involving extreme groups to illustrate, there is total effect of 2.12 (95% CI: 1.45 - 2.79) points in the SRQ-20 scores when comparing women ten points apart in PSYIPV, which is only slightly smaller than the direct effect of 2.27 (95% CI: 1.34 - 3.20) regarding PHYIPV. Also, note that putative antecedent variables (STRESS, INSTAB, UNDR5, DRUGS and ALC) showed a direct link to PSYIPV, whereas only alcohol and drugs did so on PHYIPV.

**Table 3:** **Path estimates and model fit indices of the final model shown in Figure**2**(N = 810)**

**Path**			***β *** **(95% CI)**		
PSYIPV → CMD			0.112 (0.030 - 0.194)		^**^
PHYIPV → CMD			0.227 (0.134 - 0.321)		^***^
STRESS → CMD			1.145 (0.877 - 1.413)		^***^
*F*_*SEP*_ → CMD			−0.982 (−1.294 - –0.669)		^***^
PSYIPV → PHYIPV			0.440 (0.386 - 0.494)		^***^
ALC → PHYIPV			0.129 (0.067 - 0.192)		^*^
DRUGS → PHYIPV			1.239 (1.095 - 1.384)		^**^
ALC → PSYIPV			0.143 (0.022 - 0.263)		^***^
DRUGS → PSYIPV			0.667 (0.271 - 1.062)		^***^
STRESS → PSYIPV			0.557 (0.297 - 0.817)		^***^
INSTAB → PSYIPV			1.090 (0.719 - 1.462)		^***^
UNDR5 → PSYIPV			0.429 (0.127 - 0.730)		^***^
STRESS → ALC			0.379 (0.222 - 0.536)		^***^
INSTAB → ALC			0.676 (0.476 - 0.876)		^***^
UNDR5 → ALC			−0.355 (−0.557 - –0.154)		^***^
*F*_*SEP*_ → ALC			−0.496 (−0.733 - –0.258)		^***^
STRESS → DRUGS			0.059 (0.009 - 0.108)		^*^
AGE → DRUGS			−0.010 (−0.018 - –0.003)		^**^
INSTAB → DRUGS			0.183 (0.127 - 0.240)		^***^
UNDR5 → DRUGS			−0.094 (−0.162 - –0.025)		^**^
*F*_*SEP*_ → DRUGS			−0.160 (−0.232 - –0.088)		^***^
*F*_*SEP*_ → STRESS			−0.188 (−0.263 - –0.114)		^***^
*F*_*SEP*_ → AGE			0.915 (0.380 - 1.449)		^**^
AGE → INSTAB			−0.041 (−0.056 - –0.026)		^***^
*F*_*SEP*_ → INSTAB			−0.312 (−0.443 - –0.182)		^***^
*F*_*SEP*_ → UNDR5			−0.455 (−0.591 - –0.319)		^***^
BCEC → *F*_*SEP*_**†**			0.512 (0.434 - 0.591)		^***^
HOUSE → *F*_*SEP*_**†**			0.694 (0.624 - 0.764)		^***^
EDUC → *F*_*SEP*_**†**			0.582 (0.506 - 0.657)		^***^
**Model Adjustment**					
RMSEA	0.042	(90% CI: 0.031 - 0.053); *p* ≤ .05 = 0.883			
CFI	0.967				
TLI	0.942				

*p-value < 0.05 ; **p-value < 0.01; ***p-value < 0.001.

**†**Fully standardized estimates.

Note: see Table 1 for a full description of variables.

The direct paths from alcohol misuse (ALC) and/or illicit drug abuse (DRUGS) to common mental disorders were dropped along the modeling process since they showed little significance once the other variables in the system were accounted for (*p*_{ALC → CMD}_ = 0.399 and *p*_{DRUGS → CMD}_ = 0.511). Yet, even though there is no apparent direct relationship with CMD when antecedent events are controlled for, both are by themselves risk factors for any type of IPV. Thus, a connection still exists, the association of substance misuse or abuse on CMD now running through IPV.

Focusing on the more distal events, note that most ‘propositional’ paths stemming from the socio-economic factor (*F*_*sep*_) were retained in the ‘final’ model. Table 3 shows a direct effect on CMD of −0.98 (95% CI: −1.29 - –0.67), but the total effect increases by 49% to −1.46 (95% CI: −1.79 - −1.14) when considering all pathways. Coefficients are consistently negative showing that besides less CMD, there is also less substance misuse/abuse and fewer deleterious stressors (life events, teenage pregnancy, conjugal instability and maternal burden) as the socio-economic index —the latent score— raises. Conspicuously, though, the socio-economic determination on both types of IPV seems to be fully carried through mediating processes (stressors and substance misuse/abuse). In passing, note that the relative strengths of the indicators (manifests) used to summarize *F*_*sep*_, albeit significant, are not the same. Noticing that estimates are standardized (to circumvent discrepant metrics and thus unstandardized coefficients that are difficult to compare), the indicator based on household conditions (HOUSE) seems to be the most discriminant.

Former (during pregnancy) and cumulative life events (STRESS) —death of kinship, job loss, eviction and financial worries— seem to contribute to increasing symptoms of CMD. As shown in Figure 2, all but one path remained in the ‘final’ model, conveying not only that there is an important total effect running directly to CMD (1.14; 95% CI: 0.88 - 1.41), but that substance misuse and IPV also play their part on the total effect of 1.31 (95% CI: 1.03 - 1.59). The presence of children to care for (UNDR5) runs independently from STRESS, yet shows a different pattern since its connection with CMD is fully mediated by other variables in the model.

Once the other events are taken into account, neither teen pregnancy seems to have some direct effect on CMD, nor is there any mediation by the two types of IPV and alcohol misuse. It looks as though the effect of teenage pregnancy/youth on the outcome (CMD) runs through the cumulative abuse of illegal drugs, irrespective of the presence of conjugal instability with which it is explicitly connected (AGE → INSTAB). Similarly to maternal burden (UNDR5), conjugal instability (INSTAB) failed to show a direct link to maternal CMD, but its effect runs through both types of IPV and substance consumption.

## Discussion

This study reinforces previous findings on the high magnitudes of alcohol misuse, illicit drugs consumption, intimate partner violence and common mental disorders among subjects attending public primary health care acilities in low and middle income countries [12,14], Brazil being among those [78,79]. Planning and implementing public health policies to address these issues are thus urgently required, including specific actions directed to the postnatal periods [12,14].

A better understanding on how socio-economic determinants and CMD effectively interconnect may be auspicious in this respect. Although some studies had already shown an association of IPV and other psychosocial risk factors with depression and anxiety following childbirth [19,20,22,41,80], to the best of the authors’ knowledge, the present findings are the first to address this interconnectedness within a plausible and broad framework.

### Intimate partner violence and maternal mental health

As mentioned before, comprehensive research jointly addressing IPV and women’s mental health is still scarce [27] and the knowledge on the role that psychological and physical IPV play on CMD after childbirth has been far from consistent. While there are effectively some studies showing psychological [35,37,42], physical [38] or both types of IPV [41] as risk factors for maternal depression and anxiety, research suggesting a significant role of both forms of IPV are generally based on pooled variables or on disjoint models, which fail to properly evaluate their link.

According to the present findings, both manifestations of IPV have an important and quantitatively similar effect on the occurrence of depression or anxiety symptoms during the postpartum period. However, the respective patterns are quite different. Whereas physical IPV bears its full strength in directly influencing the postnatal mental health, psychological IPV seems to be partially running through physical IPV as a mediator. From a theoretical perspective, this seems plausible. For one, the literature has consistently pointed to a pattern of progressivity in the occurrence of IPV [81]. Commonly, IPV begins with sporadic acts of minor psychological aggressions that evolve to more severe forms of violence. In some cases, psychological violence triggers reactive and thoughtless physical acts that would otherwise not occur in its absence. A higher risk of CMD symptoms following this progression is also plausible. In effect, there is recent evidence that the probability of mothers developing depression and anxiety in the months following childbirth raises as the intensity of previous IPV and other social stressors also increase [22].

### Other psychosocial covariates

The current findings may also help in understanding how socio-economic and some psychosocial conditions impact on maternal psychological status. Our results show that most relationships with CMD go through a cascade of mediating events. Recalling Figures 1 and 2, from many potential paths running directly to CMD, only four end up on this distal node. Besides both types of IPV mentioned before, direct links are exclusively observed from the socio-economic factor and stressful life events occurring during pregnancy. The effect of teenage pregnancy, conjugal instability and maternal burden apparently happens solely through substance use, be it alcohol misuse, illicit drug abuse, or both in tandem. Moreover, the effect of the latter on CMD seems to be entirely mediated through both types of IPV.

Studies based on traditional analyses have suggested a small effect of a person’s socio-economic position on postpartum depression [19,82,83]. The same would have been inferred here too had only the direct path been taken into account (4th row in Table 3). However, the underestimation of the role played by the social and economic placement of a family is redressed when considering other indirect paths. According to our model that overtly avoids treating the other variables in the system exclusively as confounders but as mediators as well, the total effect rises sharply by almost 50%. Results thus emphasize that socio-economic position poses an important strain on maternal mental health during the post-partum period and that there are several effectors involved in the process. Although maternal age, marital status and the number of children under five at home have been addressed in studies on pregnancy and postpartum depression [19,82,83], results have suggested weak or even non-significant relationships with depression and anxiety after birth [19,20]. Once again, these results may be due to the lack of an explicit assessment of indirect mediating effects. While youth and teenage pregnancy, marital instability and maternal burden arising from high parity may not be strong enough risk factors *per se* to directly kindle postpartum CMD, they make up a fertile ground wherein deleterious habits and behaviors such as abuse of illicit drugs, excessive alcohol consumption or conjugal conflicts are facilitated. These, in turn, may well lead to depression and/or anxiety in a young mother expected to care fittingly for her newborn baby.

### Strengths and limitations

The results of this study must be seen in the light of their strengths and weakness. Some methodological options stand on the positive side. The proposed theoretical model encompasses some of the most important psychosocial determinants of mental health in women [12,14,19,20] and the quality of information was assured by employing well known and comprehensive measurement tools already adapted for use in Brazil.

The statistical approach may also have contributed positively given the benefits of structural equation models as outlined in the Introduction. Yet, although structural equation models, in principle, assume implicitly a temporal ordering of relationships, the cross-sectional approach used in the current study should not be overlooked. Admittedly, a longitudinal study would have allowed to directly handling and exploring the temporality of events. Nevertheless, some ethical issues would have emerged, which the cross-sectional approach may have mitigated. In a prospective study, the identification of IPV would necessarily require action in an attempt to alleviate, reduce or even interrupt it, and thus inevitably influence the exposure-outcome relation.

The cross-sectional approach may have also influenced the recall of antecedent events, as for instance, ‘mood-sways’ in CMD-positive women modifying the perception of social support. Although recognized as an important factor related to poor mental health [14,84], we thus opted to avoid using social support in the analysis. As opposed to the other events that were measured through factual indicators, its awareness hinges on quite subjective current-state feelings and interpretations [85]. Ethical issues aside, a prospective design would therefore be optimal for suitably studying social support.

Another issue that could have potentially influenced the direction of the results concerns the use of a screening tool rather than a standardized clinical interview. Because the SRQ-20 is more sensitive to recent changes in psychological function and includes those with milder symptoms or transient psychological disturbance, one could expect some degree of false positives among the ‘true cases’ of CMD. Yet, this overestimation of true cases would only bring about bias to the effect measures of interest if it occurred differentially across IPV strata, which is not very likely.

As in any theoretically based modeling procedure, there is always scope for debate as to which phenomenon/event should have been further incorporated to the analytical model and where it should have been placed. One such example concerns sexual IPV, which might have been considered, not only for its importance in the explanatory context of mental disorders as a whole, but because its omission may have introduced some residual confounding to the exposure-outcome relations of interest. Since sexual IPV is often positively associated to both psychological/physical IPV and CMD, the present estimates are thus likely inflated provided confounding effectively took place.

Debating the external validity of results is also relevant. In view of the subjects’ characteristics, current findings seem applicable to populations commonly assisted in public primary health care units in Brazil and, possibly, other settings (countries) presenting similar socio-economic and cultural profiles. Still, some mothers under stronger stressors and severe depression, for instance, may have been missed in the study since they are more prone to social isolation and irregular visits to medical facilities. This type of selection bias may have attenuated the results since the inclusion of women scoring higher on the exposures and outcome would have given rise to stronger associations. On this account, the real situation may be even more pungent.

### Future directions

The theoretical model underlying the analysis is still propositional and requires corroboration in forthcoming studies. Although based on solid literature, the outlined relationships must still be viewed as tentative and awaiting further testing. New studies would be justified to not only corroborate or refute the current model, but also to test whether the present findings are truly generalizable (as contended here) or if there are some modification effects by domain due to social, cultural or other factor specific to Brazil. Additionally, further research should also look into how knowledge on the psychosocial determinants of mood disorders following childbirth may be used to plan and implement effective measures targeting primary and secondary prevention of postpartum psychological disturbances [86-88]. This would come in hand with recent protocols suggesting systematic approaches for postpartum depression [89,90], including the identification and management of women/couples reporting IPV [91], alcohol misuse [92] and other deleterious social health issues [22].

## Conclusion

The findings of this study reinforce the importance of IPV and other associated psychosocial characteristics as risk factors to common mental disorders following birth. In an attempt to mitigate or possibly prevent post-partum CMD, besides acknowledging socio-economic differentials, important leads may come from knowing that a woman is under stress arising from a dysfunctional relationships where IPV is extensive and constant; that she and/or her partner misuse alcohol or consume illegal drugs; and that there are several undesirable concurrent situations taking place such as teenage pregnancy, conjugal instability and/or house work overload due to many children needing to be taken care of. It is thus essential that health professionals become aware to the events accompanying CMD, particularly those of the psychosocial sphere. This could help them offer a more comprehensive assistance to mothers during the postpartum period, and by extension, allow them taking better care of their infants.

## Abbreviations

CMD: Common mental disorders; IPV: Intimate partner violence; DAG: Direct acyclic graph; BCEC: Brazilian criterion of economic classification; HOUSE: Household conditions; EDUC: Women’s schooling; STRESS: Stressful live events; AGE: Women’s age (at the beginning of the latest) pregnancy; INSTAB: Conjugal instability; UNDR5: Number of children under five; ALC: Alcohol misuse; TWEAK: Tolerance; worried; eyeopener; amnesia; K/Cut-down; CAGE: Cut-down; annoyed; guilty; eye-opener; NSDUQ: Non-student drugs use questionnaire; DRUGS: Illicit drugs abuse; CTS2: Revised conflict tactics scale; PSYIPV: Psychological IPV; PHYIPV: Physical IPV; SRQ-20: Self-reporting questionnaire; WLSMV: Weighted least squares mean and variance adjusted estimator; RMSEA: Root mean square error of approximation; CFI: Comparative fit index; TLI: Tucker-lewis index; MI: Modification indices.

## Competing interests

The authors declare that they have no competing interests.

## Authors’ contributions

Author MER managed funds for the Project, designed the study, wrote the protocol, supervised the data collection process, undertook the statistical analysis and collaborated in writing (first author) the final draft of the manuscript. Author CLM managed funds for the Project, designed the study, wrote the protocol, supervised the data collection process and collaborated in writing the final draft of the manuscript. Author CSL collaborated in the literature review, and collaborated in writing the final draft of the manuscript. Author GL collaborated in designing the study and writing the protocol, coordinated the data collection process, and assisted in writing the final draft of the manuscript. All authors read and approved the final manuscript.

## Pre-publication history

The pre-publication history for this paper can be accessed here:

http://www.biomedcentral.com/1471-2458/14/427/prepub
